# Efficacy of Intraoperative Blood Salvage in Cerebral Aneurysm Surgery

**DOI:** 10.3390/jcm10245734

**Published:** 2021-12-07

**Authors:** Bedjan Behmanesh, Florian Gessler, Elisabeth Adam, Ulrich Strouhal, Sae-Yeon Won, Daniel Dubinski, Volker Seifert, Juergen Konczalla, Christian Senft

**Affiliations:** 1 Department of Neurosurgery, University Hospital Frankfurt, Goethe University, 60528 Frankfurt am Main, Germany; florian.gessler@kgu.de (F.G.); sae-yeon.won@kgu.de (S.-Y.W.); daniel.dubinski@kgu.de (D.D.); volker.seifert@kgu.de (V.S.); juergen.konczalla@kgu.de (J.K.); christian.senft@kgu.de (C.S.); 2 Department of Anaesthesiology, Intensive Care Medicine and Pain Therapy, Goethe University, 60528 Frankfurt am Main, Germany; Elisabeth.Adam@kgu.de (E.A.); ulrich.strouhal@kgu.de (U.S.)

**Keywords:** intraoperative cell salvage, cerebral aneurysms, surgery

## Abstract

Background. The use and effectiveness of intraoperative cell salvage has been analyzed in many surgical specialties. Until now, no data exist evaluating the efficacy of intraoperative cell salvage in cerebral aneurysm surgery. Aim. To evaluate the efficacy and cost effectiveness of intraoperative cell salvage in cerebral aneurysm surgery. Methods. Data were collected retrospectively for all the patients who underwent cerebral aneurysm surgery at our institution between 2013 and 2019. Routinely, we apply blood salvage through autotransfusion. The cases were divided into a ruptured cerebral aneurysm group and a unruptured cerebral aneurysm group. Results. A total of 241 patients underwent cerebral aneurysm clipping. Of all the cerebral aneurysms, 116 were ruptured and 125 were unruptured and clipped electively. Age, location of the aneurysm, postoperative red blood cell count, intraoperative blood loss, and number of allogenic blood cell transfusions were statistically significantly different between the groups. The autotransfusion of salvaged blood could only be facilitated in eight cases with ruptured cerebral aneurysms and in none with unruptured cerebral aneurysms clipped electively (*p* < 0.01). Additionally, 35 patients with ruptured cerebral aneurysms and one patient with unruptured cerebral aneurysm required allogenic red blood cell transfusion after surgery, and 71 vs. 2 units of blood were transfused (*p* < 0.0001). In terms of cost effectiveness, a total of EUR 45,189 in 241 patients was spent to run the autotransfusion system, while EUR 13,797 was spent for allogenic blood transfusion. Conclusions. The use of cell salvage in patients with unruptured cerebral aneurysm, undergoing elective surgery, is not effective.

## 1. Introduction

Intraoperative cell salvage is widely used in many surgical specialties. Cell salvage seems to be efficacious in reducing the need for allogeneic red blood cell (RBC) transfusion in adults, and also in pediatric elective surgical procedures [1,2,3,4,5,6]. Although some studies report the advantage of using cell salvage in spine surgery, little evidence exists to support the use of intraoperative cell salvage in intracranial surgical procedures [7,8,9,10,11]. Overall, cohort studies including all types of neurosurgical procedures have reported rates of allogeneic transfusion ranging between 10 and 45% [12,13,14]. The rate of transfusion also varies depending on the procedure, from 10% in complex skull base neurosurgical procedures to 36% in patients with TBI, and as high as 45% in pediatric craniosynostosis surgery [13,15,16,17]. In patients undergoing open neurosurgical intervention for intracranial aneurysm, the occurrence of major intraoperative morbidity and complications was an independent risk factor for perioperative red blood cell (RBC) transfusion [18].

The aim of the present study was to evaluate the efficacy and cost effectiveness of intraoperative cell salvage and autotransfusion in cerebral aneurysm surgery.

## 2. Materials and Methods

Data, medical history, intraoperative protocols of all patients who underwent neurosurgical clipping for cerebral aneurysm repair between 2013 and 2019 in the University Hospital Frankfurt am Main were included in this retrospective study. Patients were stratified into two groups, depending on the cause of aneurysm surgery, in a ruptured or unruptured aneurysm group. Cell salvage using an autotransfusion system (CellSaver^®^, Haemonetics Co., Signy, Switzerland) was used in all surgical cases. Pre- and postoperative hemoglobin and hematocrit, intraoperative blood loss, duration of surgery, body mass index (BMI), habitual use of anticoagulants or non-steroidal anti-inflammatory drug (NSAID), amount of autotransfused RBCs and allogenic RBC transfusion were analyzed. Patient comorbidities were assessed according to the Charlson comorbidity index (CCI) [19].

Additionally, we looked at the costs directly associated with the routine use of autotransfusion in the setting of cerebral aneurysm clipping. Before treatment, cerebral angiography was performed in all patients and a decision in favor of clipping or coiling was made interdisciplinary. Blood transfusion was carried out in accordance with the recommendations of the Federal Medical Association; blood transfusion is recommended in all patients with a hemoglobin level below 6 g/dL. An RBC transfusion is only recommended in patients with a hemoglobin level between 6 and 8 g/dL, and limited compensation, such as coronary heart disease, heart failure and cerebrovascular disease. Furthermore, if the hemoglobin level is between 6 and 8 g/dL, blood should be given if obvious transfusion triggers, such as tachycardia, hypotension, ECG signs of ischemia and lactic acidosis, which reflect anemic hypoxia, are present.

Allogenic blood donation in patients with a hemoglobin level between 8 and 10 g/dL is only recommended with signs of anemic hypoxia.

### Statistical Analysis

Comparison of important baseline characteristics and surgical parameters between the study groups was made using Fisher’s exact test for categorical variables. Tests for normal distribution were performed using the Shapiro–Wilk test. Nonparametric tests included the Mann–Whitney U and Kruskal–Wallis tests to compare groups of data that did not follow the normal distribution. *p* < 0.05 (2-tailed) was deemed significant. All calculations were made with standard commercial software (SPSS version 22, Chicago, IL, USA).

## 3. Results

In the study period, 241 patients underwent cerebral aneurysm surgery and were included in the present study. Out of all the aneurysms, 116 were ruptured prior to (emergency) surgery and 125 were unruptured, and these patients underwent elective clipping. Table 1 entails the main baseline characteristics, and the significant differences revealed regarding age, intraoperative blood loss, autologous RBC transfusion, postoperative RBC count, and amount of allogenic red blood cell transfusion between both groups.

The patients harboring an unruptured cerebral aneurysm were significantly younger compared to the patients undergoing cerebral aneurysm repair after subarachnoid hemorrhage (*p* = 0.03). The gender distribution in both groups, level of pre- and postoperative hematocrit, BMI, habitual use of anticoagulants or NSAIDs, hemoglobin level, and duration of surgery did not differ significantly. The amount of blood loss was significantly higher in patients with ruptured cerebral aneurysms compared to those with unruptured aneurysms (*p* < 0.0001). The number of comorbidities was significantly higher in patients with ruptured cerebral aneurysms (18% vs. 6.4%; *p* = 0.006). The likelihood of allogenic RBC transfusion was significantly higher in patients suffering from subarachnoid hemorrhage, undergoing cerebral aneurysm repair, compared to patients with unruptured cerebral aneurysm clipping. The autotransfusion of salvaged blood could be facilitated in eight patients with ruptured cerebral aneurysms compared to none of the patients with unruptured cerebral aneurysms (*p* = 0.007). The mean blood loss in these 8 patients was 1981.3 mL, compared to 416.4 mL in the remaining 108 patients without autotransfusion (*p* < 0.0001). The mean amount of blood salvaged and retransfused through the cell saver was 353.8 ± 226 mL. More autotransfused patients received additional RBC transfusion than those patients who were not autotransfused (*p* = 0.04). The BMI, CCI, and habitual use of anticoagulants or NSAID were not significantly different between the groups (Table 2).

### Cost Analysis

The autotransfusion system was set up in all the surgical cases. Setting up the system was warranted in all the cases of the 125 patients undergoing elective clipping for aneurysm repair,. No patient in this group required autotransfusion of RBCs. The average cost, per procedure, of running the autotransfusion system was calculated to be EUR 189. Hence, a total of EUR 23,625 was invested in these cases. In the patients with aneurysmal subarachnoid hemorrhage, blood could be salvaged and autotransfused in eight patients; the cost of running the cell saver in the remaining 108 patients was EUR 20,412. Thus, overall, EUR 44,037 was invested without regaining blood for autotransfusion. At our institution, one unit of RBC costs EUR 86; thus, EUR 6106 was spent for 71 transfused units of RBCs.

## 4. Discussion

High intraoperative blood loss and consecutive allogenic RBC transfusion are associated with severe pathological complications, such as immunomodulation, infectious and allergic complications, transfusion-associated lung injury, and circulatory overload. These complications result in an increased risk of respiratory failure, prolonged intubation, acute respiratory distress syndrome, wound infection, sepsis, cardiac events, increased length of hospital stay, and death [20]. Blood salvage strategies in individual patients, such as preoperative correction of underlying coagulopathy and anemia, have been proposed to reduce allogenic blood donation (Meybohm P. et al., 2016, Ann Surg). Moreover, the use of intraoperative cell salvage demonstrated a significantly positive effect on reducing blood transfusion [1,3,5,6,7,8,9,10,11].

The beneficial effects of the use of intraoperative cell salvage have been investigated in many surgical specialties, such cardiac surgery, vascular surgery, spine and orthopedic surgical procedures, and many others [1,2,4,5,6,8,10] (Keding 2018 World J. Surg. Oncol.). According to the results of a recent meta-analysis published by Meyboehm et al., reviewing data from 47 studies, an overall reduction in allogeneic RBC transfusion, by a relative 39%, was observed by using washed cell salvaged blood, with the most significant result in orthopedic surgery, where the use of cell salvage reduced the exposure by 57% in 15 trials [21].

In neurosurgery, the overall RBC transfusion risk is increased in patients with aneurysmal subarachnoid hemorrhage, especially when intraoperative aneurysmal rupture occurs, or in patients with a lower preoperative hemoglobin level and higher WFNS classification [15,22]. Therefore, in accordance with our institutional standard of care, the use of intraoperative cell salvage was introduced for all patients selected for microsurgical aneurysm clipping, either electively or acutely. However, the beneficial effect of cell salvage has not been investigated until now.

Unfortunately, there are a lack of scientific data evaluating the efficacy of intraoperative cell salvage in cranial neurosurgery, except one publication from 1997. The authors of this publication concluded that the use of intraoperative cell salvage was safe and decreased the amount of allogenic blood transfusion [23]. Furthermore, almost 20% of the patients required transfusion and were operated on for different pathologies, such as unruptured cerebral aneurysms, meningiomas, and other benign and malignant brain tumors. In contrast, in our study, which only comprised patients undergoing aneurysm surgery, we found that there was hardly a need for RBC transfusion in non-ruptured compared to ruptured aneurysms (approximately 1% vs. 33%, respectively). Further, 0.8% of the patients in our investigated cohort, who were undergoing elective cerebral aneurysm surgery, required allogenic blood transfusion. Therefore, a direct comparison of our patients with the previously published data is not candidly possible.

Here, we found significant differences between both groups. First of all, the age was significantly higher in patients with ruptured cerebral aneurysms, and the ruptured aneurysms were more often located in the posterior circulation. Furthermore, there was an increase in comorbidities among the patients with SAH, undergoing emergency aneurysm clipping. The body weight, and habitual use of anticoagulants and NSIAD were almost similar in both groups. The RBCs, hemoglobin level, and hematocrit level were reduced in patients suffering from SAH. The reason for this could be the initial pretreatment with a fluid donation before the first blood test and before undergoing surgery. As expected, the amount of intraoperative blood loss was significantly higher in patients with ruptured cerebral aneurysm. Autologous blood transfusion could be facilitated in only eight patients. Due to the principle of cell salvage, consisting of blood collection, separation, washing, and filtrating, only a small part of the lost blood can be collected and retransfused [24].

Despite the abovementioned advantages of cell salvage, there are many potential complications associated with this technique, such as non-immune hemolysis, air embolus, febrile non-hemolytic transfusion reactions, mistransfusion, coagulopathy, contamination with drugs, cleaning solutions and infectious agents, and incomplete washing, leading to contamination with activated leucocytes, cytokines, and other microaggregates [25].

Since the cell saver is very rarely used in aneurysm surgery, the main problems are associated with the handling of the technique, cleaning solution, and adequate transfer. Suctioning of RBCs may cause sheer stress injury, which can result in hemolysis or damaged RBCs, and, therefore, a reduction in the return of RBCs [26,27].

An important reason for the difference in the need for transfusion between both patient groups is most likely the significant volume of blood loss. Patients with ruptured aneurysms, who are undergoing repair, are at a much higher risk for substantial intraoperative blood loss due to intraoperative rupture and renewed bleeding than patients without previous subarachnoid hemorrhage. Our findings underline the assumption that the use of cell salvage is beneficial in cases with an anticipated blood loss of more than 1000 mL.

Nevertheless, high blood loss cannot be predicted. The cell saver is a safety measure and, although only used in a few cases, reduces the administration of allogenic blood. A cost analysis of safety measures is always difficult and should primarily be viewed as a necessary measure to protect the patient. The cost of cell salvage is an important consideration, and must take into account the financial and biological benefits of autologous transfusion. Previously published data remain elusive as to whether the use of cell salvage is associated with increased or decreased costs [5,7,26,27,28,29]. Reviewing our data, we conclude that the use of cell salvage only in patients with unruptured cerebral aneurysm is not effective. In elective surgery of aneurysms, there is often no significant blood loss due to the presence of proximal control of the parent artery. In patients undergoing surgery for unruptured aneurysms, EUR 23,189 was spent for less than 1% of the patients requiring transfusion, and autotransfusion could not be facilitated to spare allogenic RBC transfusion. This underscores the limited value of regular autotransfusion in this patient group.

The effectiveness of a safety measure cannot primarily be related to costs alone. It has been shown that the use of a cell saver brings benefits and, even if rarely used, leads to the saving of allogenic blood donations.

## 5. Conclusions

The use of cell salvage in patients with unruptured cerebral aneurysms, undergoing elective surgery, is not effective. Further prospective studies are necessary to evaluate the effect of cell salvage in patients with ruptured cerebral aneurysms.

## Figures and Tables

**Table 1:** Patients’ characteristics.

Variables	Ruptured	Unruptured	*p*-Value
No. of patients	116	125	
Age (mean)	55.4 ± 14.3	52 ± 11	0.003
Sex			
Male	42 (36.2%)	39 (31.2%)	0.4
Aneurysm location			
Anterior circulation	100 (86.2%)	121 (96.8%)	0.004
Posterior circulation	16 (13.8%)	4 (3.2%)	
Hunt & Hess Grade			
I	26 (22.4%)		
II	19 (16.4%)		
III	23 (19.8%)		
IV	25 (21.5%)		
V	23 (19.8%)		
Red blood cells (/pL)			
Preoperative	4.2 ± 0.4	4.5 ± 0.4	0.2
Postoperative	3.5 ± 0.5	3.8 ± 0.5	0.009
Hematocrit (%)			
Preoperative	37.6 ± 3.9	40.1 ± 4.2	0.1
Postoperative	31.4 ± 4.9	33.3 ± 4.8	0.1
Hemoglobin (g/dL)			
Preoperative	12.9 ± 1.5	13.7 ± 1.2	0.1
Postoperative	10.6 ± 1.6	11.4 ± 1.4	0.2
Median BMI	25	26	0.4
Median CCI	2	1	0.05
CCI 0-2	95 (82%)	117 (94%)	0.006
NSAID	4 (3.4%)	5 (4%)	1.0
Anticoagulation (*n*/%)	11 (9.4%)	7 (5.6%)	0.3
Intraoperative blood loss (mL)	601 ± 809	244.1 ± 197	0.00001
Operation time (min)	200 ± 53.9	201.5 ± 50.1	0.7
Allogenic RBC transfusion(*n* [%])	35 (32.4%)	1 (0.8%)	0.0001
Allogenic blood units (*n*)	71 (61.2%)	2 (1.6%)	
Autotransfusion (*n*)	8 (6.9%)	0	0.007
Costs for CS setup	€21,924	€23,625	

**Table 2:** Data of patients in the ruptured group stratified by RBC retransfusion and no retransfusion.

	Cellsaver	No Cellsaver	*p* Value
No. of patients	8	108	
Age (mean)	52.4 y	56.1 y	0.4
Sex			
Male	2 (25%)	40 (37%)	0.7
Female	6 (75%)	68 (63%)	
Anterior circulation	6 (75%)	95 (88%)	0.3
Posterior circulation	2 (25%)	14 (13%)	
Hunt & Hess Grade			
I	1 (12.5%)	25 (23.1%)	1.0
II	1 12.5%)	18 (16.7%)	1.0
III	3 (37.5%)	21(19.4%)	0.15
IV	2 (25%)	22 (20.3%)	0.7
V	1 (12.5%)	22 (20.4%)	1.0
Red blood cells (/pL)			
Preoperative	4.0 ± 0.4	4.2 ± 0.4	0.8
Postoperative	3.2 ± 0.3	3.5 ± 0.5	0.5
Hematocrit (%)			
Preoperative	37.8 ± 4.1	37.8 ± 3.8	0.2
Postoperative	29.0 ± 3.1	31.5 ± 5.0	0.4
Hemoglobin (g/dL)			
Preoperative	12.1 ± 1.5	13.0 ± 1.5	0.2
Postoperative	9.4 ± 1.3	10.7 ± 1.7	0.9
Median BMI	25	25	0.4
Median CCI	2	2	0.5
CCI 0-2	7 (87.5%)	88 (81.5%)	1.0
NSAID	0	4 (3.7%)	
Anticoagulation (*n*/%)	0	11 (10.2%)	
Intraoperative blood loss (mL)	1981.3 ± 1587.4	416.4 ± 315.0	0.00001
Operation time (min)	182.5 ± 51.1	200.8 ± 53.9	0.4
Allogenic blood cell transfusion (*n* [%])	5 (62.5%)	30 (26.9%)	0.04
Allogenic RBC units (*n*)	26	45	
Amount of autotransfusion (mL)	353.8±226	0	

## Data Availability

Data available on request from the authors.
